# ﻿Three new genera of wolf spiders (Araneae, Lycosidae) living on the forest floor in East Asia

**DOI:** 10.3897/zookeys.1239.152834

**Published:** 2025-05-21

**Authors:** Lu-Yu Wang, Yuri M. Marusik, Zhi-Sheng Zhang

**Affiliations:** 1 Key Laboratory of Eco-environments in Three Gorges Reservoir Region (Ministry of Education), School of Life Sciences, Southwest University, Chongqing 400715, China Southwest University Chongqing China; 2 Institute for Biological Problems of the North RAS, Portovaya Str.18, Magadan 685000, Russia Institute for Biological Problems of the North RAS Magadan Russia; 3 Department of Zoology & Entomology, University of the Free State, Bloemfontein 9300, South Africa University of the Free State Bloemfontein South Africa; 4 Altai State University, Lenina Pr., 61, Barnaul, RF-656049, Russia Altai State University Barnaul Russia

**Keywords:** Aranei, China, description, Japan, Korea, morphology, new combination, new genus, new species, taxonomy

## Abstract

Three new genera as well as one new species belonging to Lycosidae are described, *Houcosa***gen. nov.** (type species: *Houcosazhaoi***sp. nov.**, ♂♀), *Kuncosa***gen. nov.** (type species: *Arctosaningboensis* Yin, Bao & Zhang, 1996) and *Loongcosa***gen. nov.** (type species: *Pardosadentitegulum* Yin, Peng, Xie, Bao & Wang, 1997). Seven new combinations are proposed: *Kuncosafujiii* (Tanaka, 1985), **comb. nov.**, *K.hikosanensis* (Tanaka, 1985), **comb. nov.**, *K.kwangreungensis* (Paik & Tanaka, 1986), **comb. nov.**, *K.ningboensis* (Yin, Bao & Zhang, 1996), **comb. nov.** (all ex. *Arctosa*), *K.zhui* (Yu & Song, 1988), **comb. nov.**, *Loongcosadentitegulum* (Yin, Peng, Xie, Bao & Wang, 1997), **comb. nov.** and *L.wuyiensis* (Yu & Song, 1988), **comb. nov.** (all ex. *Pardosa*). The male of *Kuncosaningboensis* and the female of *Loongcosadentitegulum* are described for the first time. Detailed descriptions of copulatory organs and somatic features of the new and known species are provided, along with photographs and a distribution map.

## ﻿Introduction

The spider family Lycosidae Sundevall, 1833, commonly known as wolf spiders, is typically abundant in open habitats around the world (Jocqué and Alderweireldt 2005). They seldom live in forests. However, in Asia, some wolf spider species do inhabit forests, such as *Allotrochosina* Roewer, 1960 (Wang et al. 2021), *Ovia* Sankaran, Malamel & Sebastian, 2017 (Lu et al. 2018), *Sinartoria* Wang, Framenau & Zhang, 2021 (Wang et al. 2021) and *Zantheres* Thorell, 1887 (Wang and Zhang 2020). They also share a common characteristic: the first pair of legs are all white from the femur onward in males. It seems that this increases visibility during courtship in the dark forest environment.

Lycosidae ranks as the fifth largest family of spiders, comprising 135 genera and 2489 species (WSC 2025), which are classified into 10 subfamilies (Piacentini and Ramírez 2019). Artoriinae Framenau, 2007 (Framenau 2007; Framenau and Baehr 2018; Vink 2002, etc.) and some genera, such as *Acantholycosa* Dahl, 1908 (Marusik et al. 2004), *Hippasa* Simon, 1885 (Wang et al. 2015), *Pirata* Sundevall, 1833, *Piratula* Roewer, 1960 (Omelko et al. 2011) and *Xerolycosa* Dahl, 1908 (Wang et al. 2024) have been well revised in East Asia. However, the vast majority of genera, especially in large ones with extremely rich species diversity such as *Alopecosa* Simon, 1885, *Arctosa* C.L. Koch, 1847, *Hogna* Simon, 1885, *Lycosa* Latreille, 1804 and *Pardosa* C.L. Koch, 1847, still play the roles of a ‘trash can = waste basket’. For example, *Arctosafujiii* Tanaka, 1985, *A.kwangreungensis* Paik & Tanaka, 1986, *A.hikosanensis* Tanaka, 1985, *A.ningboensis* Yin, Bao & Zhang, 1996, *Pardosadentitegulum* Yin, Peng, Xie, Bao & Wang, 1997, *P.wuyiensis* Yu & Song, 1988, *P.zhui* Yu & Song, 1988 etc. are definitely misplaced in *Arctosa* and *Pardosa*.

This paper aims to sort out some wolf spiders living on forest floor from East Asia and to describe three new genera: *Houcosa* gen. nov., *Kuncosa* gen. nov. and *Loongcosa* gen. nov., as well as one new species *Houcosazhaoi* sp. nov., to establish new combinations and to describe the previously unknown male of *Kuncosaningboensis* and the female of *Loongcosadentitegulum*.

## ﻿Material and methods

All specimens are preserved in 75% ethanol and were examined, photographed, and measured using a Leica M205A stereomicroscope equipped with a Leica DFC450 camera, and LAS v. 4.6 software. Male palps and epigynes were examined and illustrated after they were dissected. Epigynes were cleared by immersing them in a pancreatin solution for about 1 h (Álvarez-Padilla and Hormiga 2007). Eye sizes were measured as the maximum dorsal diameter. Leg measurements are shown as: total length (femur, patella and tibia, metatarsus, tarsus). All measurements are given in millimeters. Specimens examined here are deposited in the Collection of Spiders, School of Life Sciences, Southwest University, Chongqing, China (**SWUC**), the Hunan Normal University (**HNU**), and the National Science Museum, Tokyo, Japan (**NSMT**).

Abbreviations used in the text: **ALE** = anterior lateral eye; **AME** = anterior median eye; **PLE** = posterior lateral eye; **PME** = posterior median eye; **RTA** = retrolateral tibial apophysis.

## ﻿Taxonomy

**Family Lycosidae Sundevall, 1833**

Common name. 狼蛛科

### ﻿ Houcosa gen. nov.

Taxon classificationAnimaliaAraneaeLycosidae

65816340-F732-5716-A4F5-A61E28DD3442

https://zoobank.org/49173A9A-1A0A-4232-A61F-FA1312A486A0

#### Type species.

*Houcosazhaoi* sp. nov.

#### Etymology.

The generic name is a compound noun derived from the ancient Chinese mythical creatures ‘hou’ (犼) and ‘-cosa’, a common ending for Lycosidae genera. The gender is feminine.

#### Diagnosis.

*Houcosa* gen. nov. is similar to *Loongcosa* gen. nov. in having a modified palp tibia, 2 pairs of spines on the male palp tibia, a biforked basoembolic apophysis (BEA, Figs 2, 12, 14), absent median apophysis and conductor, an extended posterior septum of the epigynes (Figs 3C, D, 13C, D, 15C, D), but differs by the tibia as long as cymbium (Fig. 2A–C) (vs 1/3 length, Figs 12A–C, 14A–C), the long and membranous terminal apophysis located on the anterior side of the embolus (TA, Fig. 2F) (vs short and sclerotized, located on the posterior side of the embolus; Figs 12F, 14F), the thin embolus (Fig. 2F) (vs broad, Figs 12B, D, F, 14 B, D, F), the small and semicircular tegular apophysis (TeA, Figs 2B–E) (vs absent), the triangular retrolateral tegular apophysis (RTeA, Fig. 2A–E) (vs strongly sclerotized and grooved, Figs 12B–E, 14B–E), the small and semicircular septum (Se, Fig. 3C) (vs large and almost tongue-shaped, Figs 13C, 15C), the S-shaped stalk of the spermatheca (SS, Fig. 3D) (vs arc-shaped, Figs 14D, 15D), large and spherical head of the spermatheca (HS, Fig. 3D) (vs small and spherical or elongate, Figs 14D, 15D).

**Figure 1.**
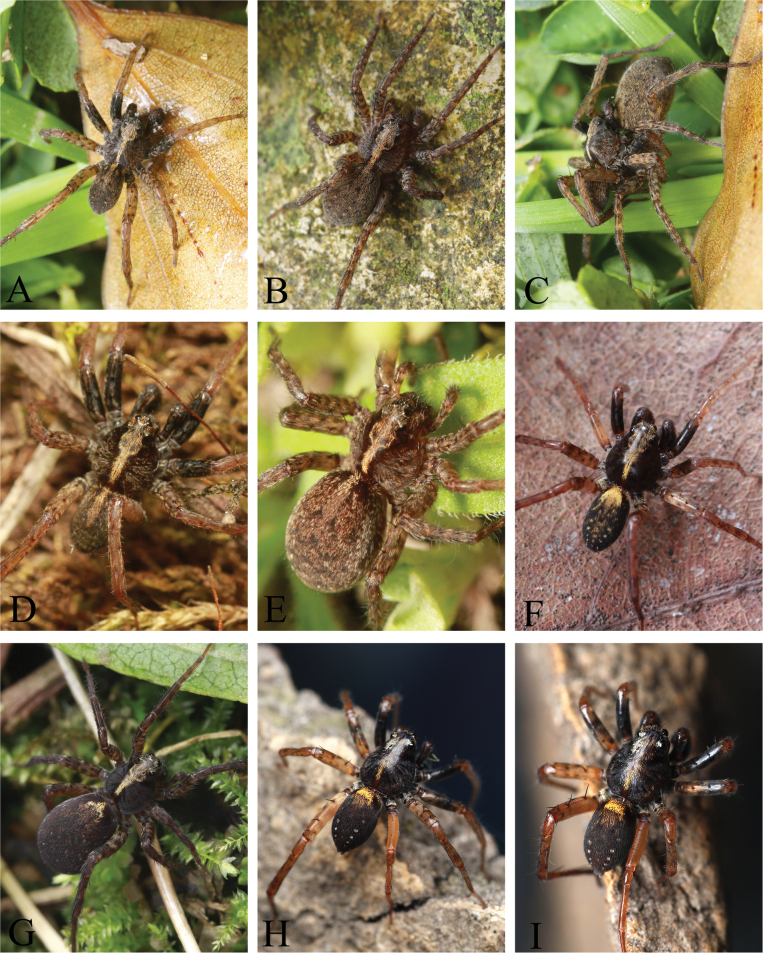
Living specimen of wolf spiders **A–C***Kuncosaningboensis* (Yin, Bao & Zhang, 1996) (**A** male, **B** female, **C** mating) **D, E***Kuncosazhui* (Yu & Song, 1988) (**D** male, **E** female) **F, G***Loongcosadentitegulum* (Yin, Peng, Xie, Bao & Wang, 1997) (**F** male, **G** female) **H, I***Loongcosawuyiensis* (Yu & Song, 1988) (male) (**A–C, F–I** photographed by Qian-Le Lu, **D–E** photographed by Luyu Wang).

**Figure 2.**
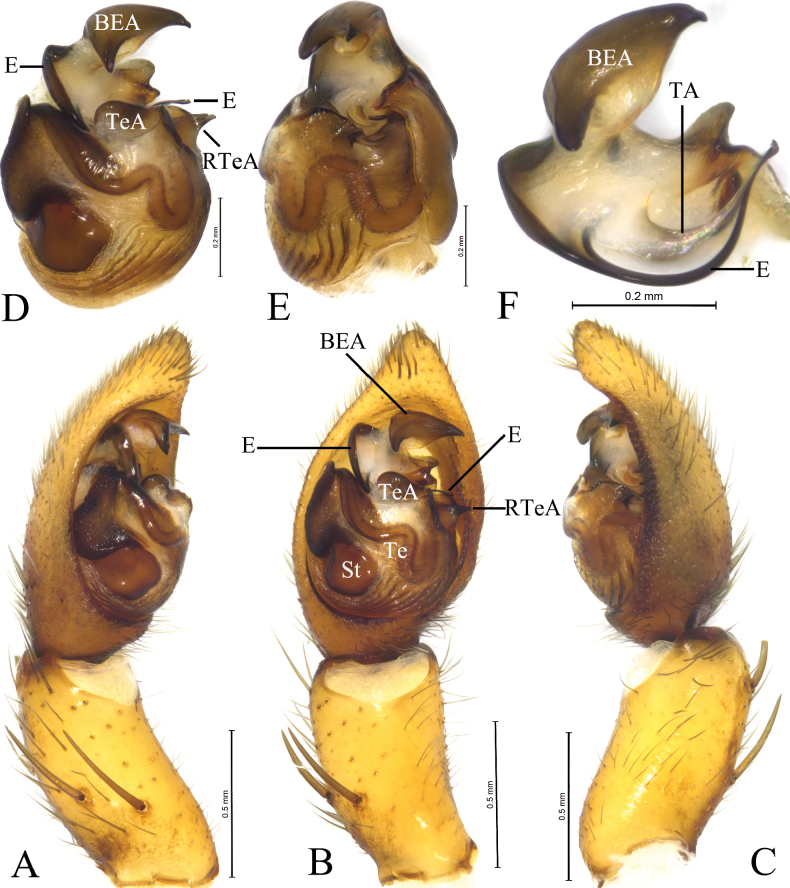
*Houcosazhaoi* sp. nov. **A–C** holotype male **D–F** paratype male **A** male left palp, prolateral view **B** same, ventral view **C** same, retrolateral view **D** left male palp, bulb, ventral view **E** same, retrolateral view **F** embolus and terminal apophysis, ventral view. Abbreviations: BEA = basoembolic apophysis; E = embolus; RTeA = retrolateral tegular apophysis; St = subtegulum; TA = Terminal apophysis; Te = tegulum; TeA = Tegular apophysis.

**Figure 3.**
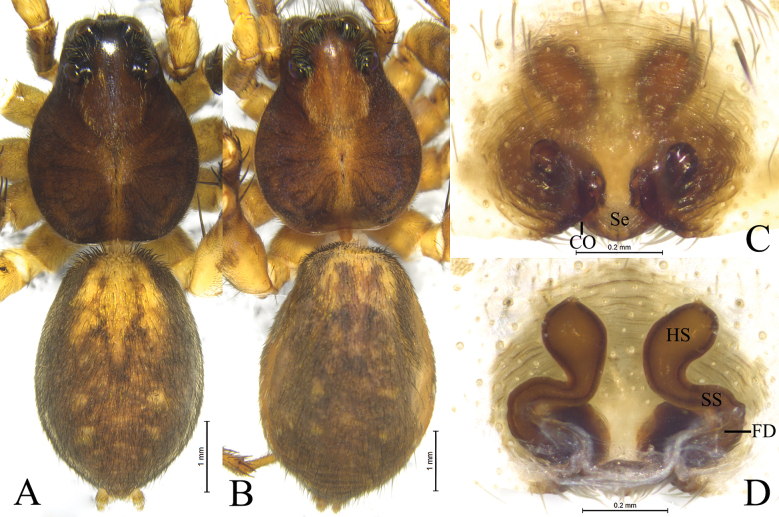
*Houcosazhaoi* sp. nov. **A** holotype, male **B–D** paratype, female **A** male habitus, dorsal view **B** female habitus, dorsal view **C** epigyne, ventral view **D** vulva, dorsal view. Abbreviations: CO = copulatory opening; FD = fertilization duct; HS = head of spermatheca. Se = septum; SS = stalk of spermatheca.

#### Description.

Carapace brown, pear-shaped. Eyes region black. Cervical groove and radial furrows indistinct (Fig. 3A, B). Chelicerae brown, with 3 teeth on both margins. Labium brown, with dark base, longer than wide. Endites brown, longer than wide. Sternum brown and scutellate, with sparse black setae. Legs brown, with black pigmentation. Leg formula: 4132 or 4123. Opisthosoma oval, dorsum black brown, with lanceolate cardiac mark in anterior half, venter brown.

***Palp*** (Fig. 2): Tibia modified, rounded ventro-proximally and dorso-distally with 2 pairs of spines: pair of dorsal and pair of prolateral spines. Cymbium with short tip, cymbial claws absent. Subtegulum (St) located postero-prolaterally. Sperm duct waved. Tegulum with distal triangular apophysis (RTeA). Tegular apophysis (TeA) small, semicircular. Conductor and median apophysis lacking. Embolic division complex, palea lacking, with large and heavily sclerotized basoembolic apophysis with 2 horned-like extensions (BEA), terminal apophysis (TA) membranous. Embolus (E) originated on dorsal part of embolic division, whip-shaped.

***Epigyne*** (Fig. 3C, D): Epigynal plate slightly wider than long, fovea absent, septum (Se) small with semicircular posterior part. Spermathecae S-shaped with long stalks (SS) and suboval heads (HS).

#### Composition.

*Houcosazhaoi* sp. nov.

#### Distribution.

China (Guangxi) (Fig. 16).

### ﻿ Houcosa zhaoi sp. nov.

Taxon classificationAnimaliaAraneaeLycosidae

B6EB9187-054E-5B6F-8547-7B51F3DF5493

https://zoobank.org/5AC153EA-7863-4621-B806-CB813F046E7A

Common name. 赵氏犼蛛 Figs 2 , 3 , 16

#### Type material.

***Holotype*** ♂ (SWUC-T-LY-19-01): China, Guangxi, Laibin City, Jinxiu Co., Pingban, 24°05'25"N, 110°10'34"E, elev. 1053 m, 26 April 2023, W.Q. Zhao leg. ***Paratypes***: 4♂5♀ (SWUC-T-LY-19-02–10), with same data as for holotype.

#### Etymology.

The specific epithet is taken from the family name of Mr Wenqi Zhao (Nanchang, China), who collected this species.

#### Diagnosis.

See genus diagnosis.

#### Description.

**Male** (holotype, Fig. 3A). Total length 6.85. Prosoma 3.21 long, 2.43 wide; opisthosoma 3.51 long, 2.43 wide. Carapace greyish brown, lacking distinct pattern, with lighter median band in posterior part. Eye sizes and interdistances: AME 0.12, ALE 0.12, PME 0.37, PLE 0.32; AME–AME 0.07, AME–ALE 0.07, PME–PME 0.30, PME–PLE 0.35. Clypeus height 0.22. Legs yellow brown, with black pigmentation. Leg measurements: I 8.69 (2.24, 3.10, 2.23, 1.12); II 8.40 (2.23, 2.89, 2.09, 1.19); III 8.63 (2.23, 2.69, 2.56, 1.15); IV 11.87 (2.84, 3.74, 3.78, 1.51). Opisthosoma oval. Dorsum greyish brown, with yellow brown markings. Venter yellow brown.

***Palp*** (Fig. 2). Tibia with 2 pairs of prolateral spines. Embolus whip-shaped. Basoembolic apophysis biforked. No conductor. Terminal apophysis long and membranous. Tegular apophysis small and semicircular. Retrolateral tegular apophysis triangular.

**Female** (paratype SWUC-T-LY-19-02, Fig. 3B). Total length 8.15. Prosoma 3.62 long, 2.79 wide; opisthosoma 4.35 long, 2.96 wide. Carapace with distinct light median band, wide in cephalic part as wide as PME–PME, and thin in posterior part. Eye sizes and interdistances: AME 0.10, ALE 0.13, PME 0.38, PLE 0.34; AME–AME 0.13, AME–ALE 0.10, PME–PME 0.32, PME–PLE 0.38. Clypeus 0.15 high. Leg measurements: I 8.70 (2.54, 3.13, 2.08, 0.95); II 8.54 (2.43, 3.12, 1.90, 1.09); III 8.18 (2.26, 2.55, 2.25, 1.12); IV 12.59 (3.08, 4.10, 3.92, 1.49).

***Epigyne*** (Fig. 3C, D). Septum slightly longer than wide with semicircular posterior part. Copulatory openings located between septum and lateral walls. Stalk of spermatheca S-shaped, head of spermatheca suboval. Fertilization ducts crescent-shaped.

#### Habitat.

Live on the forest floor of broad-leaved forests.

#### Distribution.

Currently known only from the type locality, China (Guangxi, Fig. 15).

### ﻿ Kuncosa gen. nov.

Taxon classificationAnimaliaAraneaeLycosidae

0EAE2F16-AE6F-5D45-A24C-479875FD32C2

https://zoobank.org/5D0128DB-35B5-4744-8E78-92CFCE5A7467

#### Type species.

*Arctosaningboensis* Yin, Bao & Zhang, 1996.

#### Etymology.

The generic name is a compound noun derived from the ancient Chinese mythical creatures ‘kun’ (鲲) and ‘-cosa’, a common ending for Lycosidae genera. The gender is feminine.

#### Diagnosis.

**Figure 4.**
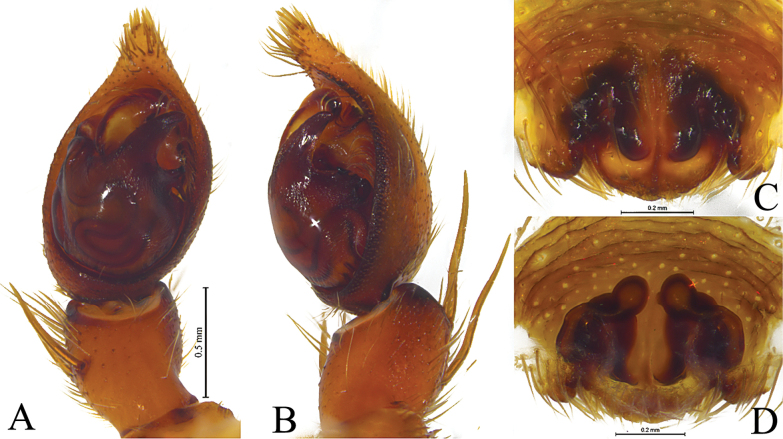
*Kuncosafujiii* (Tanaka, 1985), comb. nov. **A, B** holotype male **C, D** paratype female **A** male left palp, ventral view **B** same, retrolateral view **C** epigyne, ventral view **D** vulva, dorsal view.

**Figure 5.**
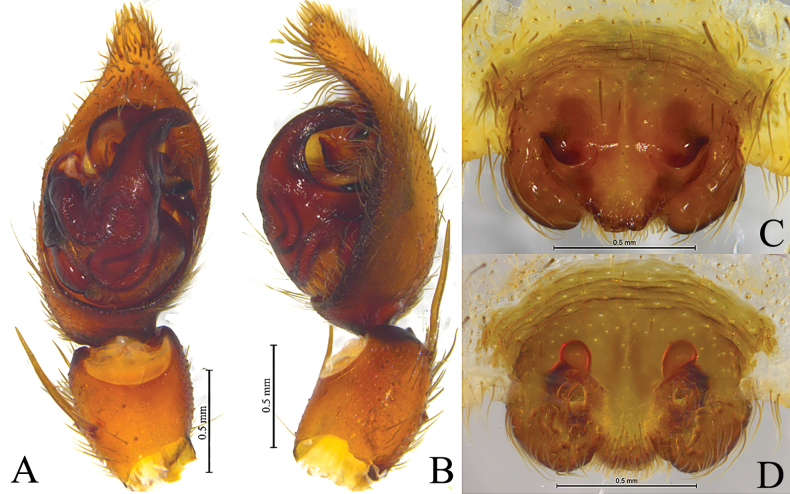
*Kuncosahikosanensis* (Tanaka, 1985), comb. nov. **A, B** holotype male **C, D** paratype femele **A** male left palp, ventral view **B** same, retrolateral view **C** epigyne, ventral view **D** vulva, dorsal view.

*Kuncosa* gen. nov. is similar to *Loongcosa* gen. nov. in having 2 pairs of spines in male palp tibia, a developed retrolateral tegular apophysis (Figs 4A, B, 5A, B, 8A–E, 10A–E, 12A–E, 14A–E), a broad septum (Figs 4C, D, 5C, D, 9C, D, 11C, D, 13C, D, 15C, D), but differs from the later by tibia as half-length of cymbium (Figs 4A, B, 5A, B, 8A–C, 10A–C) (vs 1/3 length, figs 12A–C, 14A–C), the thin embolus lacking pars pendula (Figs 8F, 10F) (vs broad with pars pendula, Figs 12B, D, F, 14B, D, F), the comma-shaped basoembolic apophysis (BEA, Figs 8F, 10F) (vs bifurcated, Figs 12A–F, 14A–F), the strong and triangular terminal apophysis (TA, Figs 8F, 10F) (vs thin, and finger-shaped, Figs 12F, 14F), the tegular apophysis present (TeA, Figs 4A, B, 5A, B, 8A–E, 10A–E) (vs absent), the bifurcated retrolateral tegular apophysis (RTeA, Figs 4A, B, 5A, B, 8A–E, 10A–E) (vs strong sclerotized and grooved, Figs 12B–E, G, 14B–E, G), truncated posterior margin of septum (Figs 4C, D, 5C, D, 7, 9C, D, 11C, D) (vs with a tongue like apophysis, Figs 13C, D, 15C, D).

**Figure 6.**
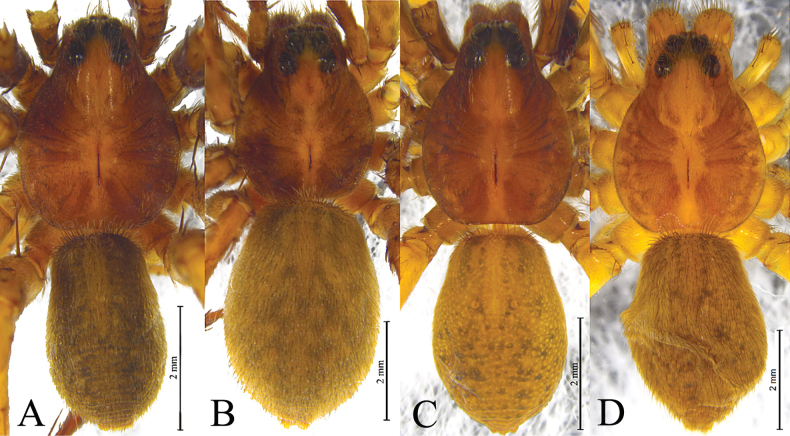
*Kuncosa* spp. **A, B***Kuncosafujiii* (Tanaka, 1985) **C, D***Kuncosahikosanensis* (Tanaka, 1985) **A, C** holotype male habitus, dorsal view **B, D** paratype female habitus, dorsal view.

**Figure 7.**
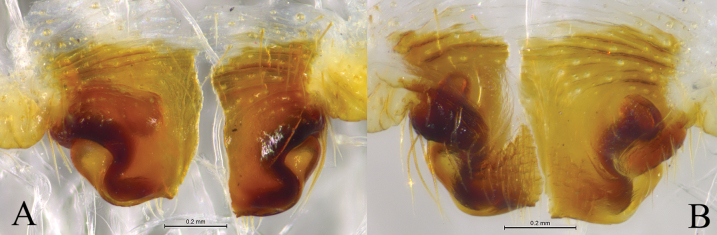
*Kuncosaningboensis* (Yin, Bao & Zhang, 1996), comb. nov. holotype, female **A** epigyne, ventral view **B** vulva, dorsal view.

#### Description.

Carapace brown with light median band. Eyes region black. Fovea vertical. Cervical groove and radial furrows indistinct. Chelicerae elongate, brown, with 3 teeth on both margins. Labium yellow brown, with dark base. Endites yellow brown. Sternum brown and scutellate, with sparse black setae. Legs brown, with black pigmentation. Leg formula: 4132. Opisthosoma oval. Dorsum dark brown, with lanceolate cardiac mark in anterior half, and black irregular markings in posterior half. Venter brown.

**Figure 8.**
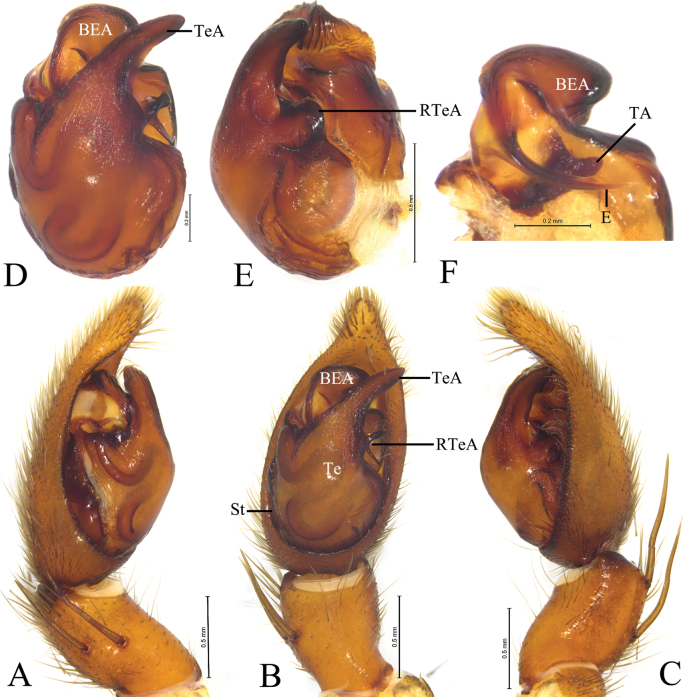
*Kuncosaningboensis* (Yin, Bao & Zhang, 1996), comb. nov. from Tianmu Mt. **A** male left palp, prolateral view **B** same, ventral view **C** same, retrolateral view **D** left male palp, bulb, ventral view **E** same, retrolateral view **F** embolus and terminal apophysis, ventral view. Abbreviations: BEA = basoembolic apophysis; E = embolus; RTeA = retrolateral tegular apophysis; St = subtegulum; TA = Terminal apophysis; Te = tegulum; TeA = Tegular apophysis.

***Palp*** (Figs 4A, B, 5A, B, 8, 10): Tibia with 2 pairs of spines, and rounded disto-dorsal edge. Cymbium asymmetric, with short tip and lacking claws. Sperm duct weavy. Tegulum with long tegular apophysis (TeA) and short bifurcate retrolateral apophysis (RTeA). Conductor and median apophysis lacking. Embolic division complex, palea absent, terminal apophysis (TA) triangle- or fingernail-like, Basoembolic apophysis (BEA) strongly sclerotized Embolus needle-shaped, originated from proventral side, lacking pars pendula and with abrupt tip.

**Figure 9.**
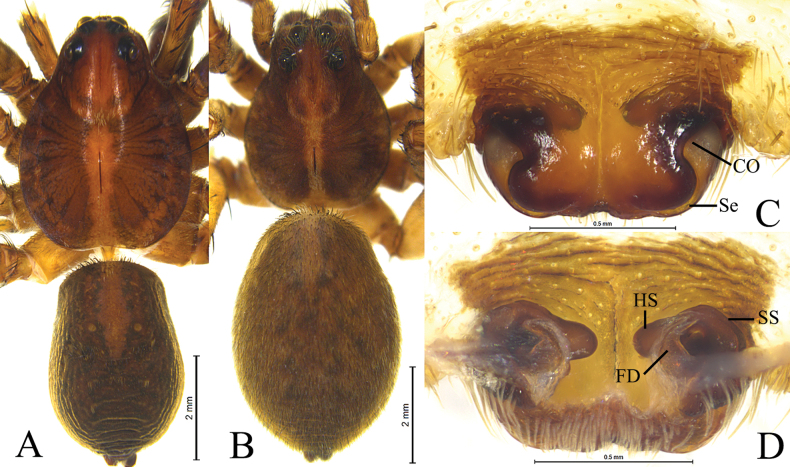
*Kuncosaningboensis* (Yin, Bao & Zhang, 1996), comb. nov. from Tianmu Mt. **A** male habitus, dorsal view **B** female habitus, dorsal view **C** epigyne, ventral view **D** vulva, dorsal view. Abbreviations: CO = copulatory opening; FD = fertilization duct; HS = head of spermatheca. Se = septum; SS = stalk of spermatheca.

***Epigyne*** (Figs 4C, D, 5C, D, 7, 9C, D, 11C, D): Epigynal plate wider than long, fovea indistinct, covered by broad septum and oval. Stalk of spermatheca (SS) C- or S-shaped, head of spermatheca (HS) suboval.

#### Composition.

*Kuncosafujiii* (Tanaka, 1985), *K.hikosanensis* (Tanaka, 1985), *K.kwangreungensis* (Paik & Tanaka, 1986), *K.ningboensis* (Yin, Bao & Zhang, 1996) and *K.zhui* (Yu & Song, 1988).

#### Distribution.

China (Anhui, Chongqing, Guizhou, Hubei, Hunan, Jiangxi, Jiangsu, Zhejiang), southern part of Japan and Korea. (Fig. 16)

#### Remarks.

Although the types of *Arctosakwangreungensis* Paik & Tanaka, 1986 were not examined, it is clear from the descriptions in Paik and Tanaka (1986) that this species belongs to the *Kuncosa*.

### ﻿ Kuncosa fujiii

Taxon classificationAnimaliaAraneaeLycosidae

(Tanaka, 1985) comb. nov.

B35E2655-0BCB-5C80-A3A1-D2C3DDEA4665

Figs 4 , 6A, B , 16

Arctosa fujiii Tanaka, 1985: 57, figs 9–12 (♂♀); Chikuni 1989: 112, fig. 15 (♂♀); Tanaka 1991: 296, figs 9–12 (♂♀); Tanaka 2009: 232, figs 56, 57 (♂♀).

#### Material examined.

1♂ (***paratype***) and ♀ (***holotype***), Japan, Honshu, Saitama Pref., Hidaka-cho, 21 April 1973, H. Tanaka leg. (NSMT).

#### Diagnosis.

This species is similar to *K.hikosanensis* (Fig. 5) but differs from the latter by the thin and short retrolateral tegular apophysis (RTeA, Fig. 4A, B) (vs thick and long), and the semicircular septum (Se, Fig. 4C) (vs almost triangular).

#### Description.

See Tanaka (1985, 2009). Habitus, male palps and epigynes as in Figs 4, 5A, B.

#### Arguments for transferring.

Similar to the type species: tegulum with long median apophysis (TeA) lacking in other lycosid genera.

#### Distribution.

Japan (Fig. 16).

### ﻿ Kuncosa hikosanensis

Taxon classificationAnimaliaAraneaeLycosidae

(Tanaka, 1985) comb. nov.

59F9C141-E34C-5BBE-B23C-28DB64EA8A55

Figs 5 , 6C, D , 16

Arctosa hikosanensis Tanaka, 1985: 59, figs 13–16 (♂♀); Tanaka 1991: 299, figs 13–16 (♂♀); Tanaka 2009: 232, figs 58–59 (♂♀); Serita 2019: 150, fig. 1f–g (♂♀).

#### Material examined.

1♂ (***paratype***) and ♀ (***holotype***), Japan, Kyushu, Fukuoka Prefecture, Hikosan, 18–19 April 1979, T. Goto leg. (NSMT).

#### Diagnosis.

See the diagnosis of *K.fujiii* (Tanaka, 1985).

#### Description.

See Tanaka (1985). Habitus, male palps and epigynes as in Fig. 5.

#### Arguments for transferring.

Similar to the type species: tegulum with long median apophysis (TeA) lacking in other lycosid genera.

#### Distribution.

Japan (Fig. 16).

### ﻿ Kuncosa ningboensis

Taxon classificationAnimaliaAraneaeLycosidae

(Yin, Bao & Zhang, 1996) comb. nov.

E6B0BDE9-C085-523D-A723-44D3BFF26E6D

Common name. 宁波鲲蛛 Figs 1A–C , 8 , 9 , 16

Arctosa ningboensis Yin et al., 1996: 5, figs 1–3 (♀); Yin et al. 1997b: 98, fig. 45a–c (♀); Song et al. 1999: 319, fig. 189H (♀); Dong 2018: 60, figs 9–3. A–C, pl. 13 (♀).

#### Material examined.

**China**, **Anhui**: 1♀,Huangshan City, Xiuning Co., Qiyun Mt., 29°48'50"N, 118°02'43"E, elev. 168 m, 7 April 2012, L.Y. Wang and X.K. Jiang leg. (SWUC) • 5♂ 2♀, Qiyun Mt., Dongtianfudi, 29°48'25"N, 118°02'29"E, elev. 441 m, 23 October 2013, L.Y. Wang, et al. leg. (SWUC) • **Zhejiang**: 1♀ (**holotype**), Ningbo City, 29.9°N, 121.5°E, 20 May -5 June 1987, Y.J. Zhang leg. (HNU) • 1♂ 1♀, Ningbo City, Beicang Distr., 29°49'52"N, 121°51'57"E, elev. 89 m, 29 March 2024, Q.L. Leg. (SWUC) • 90♂61♀, Hangzhou City, Lin’an Co., Tianmu Mt., 30°19'10"N, 119°26'52"E, elev. 382 m, 9 April 2012, L.Y. Wang leg. (SWUC) • 1♀, Tianmu Mt., 30°18'43"N, 119°26'53"E, elev. 335 m, 9 April 2012, L.Y. Wang leg. (SWUC) • 1♂, Tianmu Mt., 30°18'51"N, 119°26'25"E, elev. 346 m, 21 April 2011, Z.X. Li and L.Y. Wang leg. (SWUC) • 12♂16♀, Tianmu Mt., 30°19'05"N, 119°26'10"E, elev. 351 m, 23 April 2011, Z.X. Li and L.Y. Wang leg. (SWUC).

#### Diagnosis.

**Figure 10.**
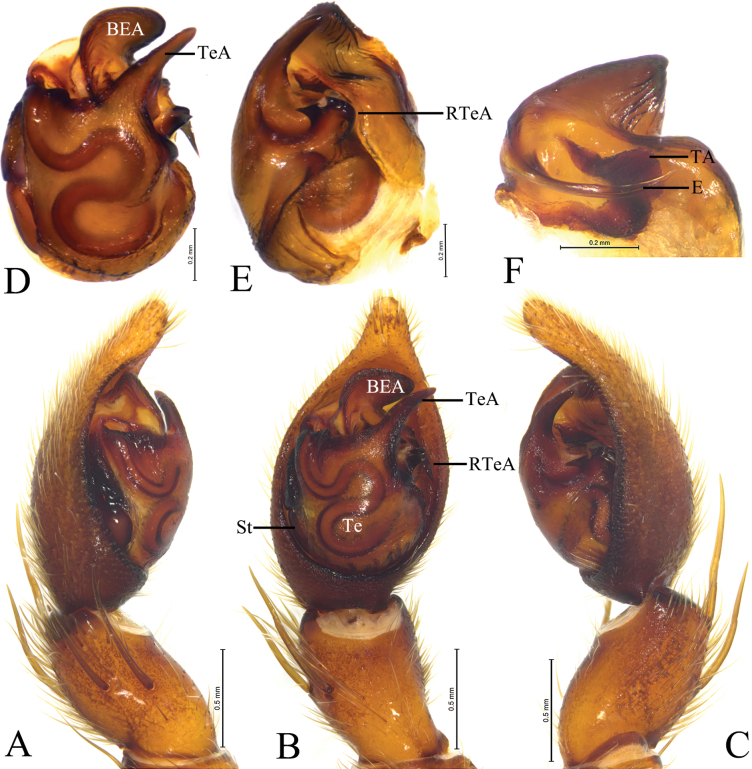
*Kuncosazhui* (Yu & Song, 1988), comb. nov. from Simian Mt. **A** male left palp, prolateral view **B** same, ventral view **C** same, retrolateral view **D** left male palp, bulb, ventral view **E** same, retrolateral view **F** embolus and terminal apophysis, ventral view. Abbreviations: BEA = basoembolic apophysis; E = embolus; RTeA = retrolateral tegular apophysis; St = subtegulum; TA = Terminal apophysis; Te = tegulum; TeA = Tegular apophysis.

**Figure 11.**
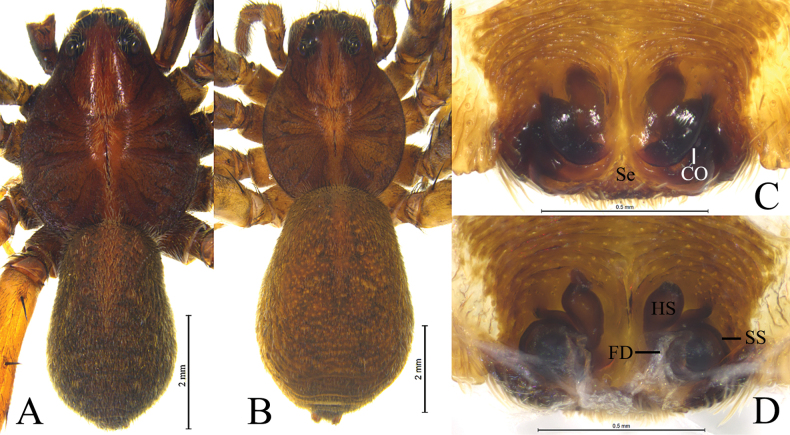
*Kuncosazhui* (Yu & Song, 1988), comb. nov. from Simian Mountain **A** male habitus, dorsal view **B** female habitus, dorsal view **C** epigyne, ventral view **D** vulva, dorsal view. Abbreviations: CO = copulatory opening; FD = fertilization duct; HS = head of spermatheca. Se = septum; SS = stalk of spermatheca.

This species is similar to *K.zhui* in having hook-like posterior arm of the retrolateral tegular apophysis and a spherical head of the spermatheca (Figs 8B–E, 9D, 10B–E, 11D), but differs from the latter by the thick tegular apophysis (TeA, Fig. 8A–E) (vs thin, Fig. 10A–E), the almost triangle-like terminal apophysis (TA, Fig. 8F) (vs fingernail-like, Fig. 10F), the digital-like anterior arm of retrolateral tegular apophysis in retrolateral view (RTeA, Fig. 8B–E) (vs triangular-like, Fig. 10B–E), the semicircular copulatory openings (CO, Fig. 9C) (vs slit-like, Fig. 11C), and the C-shaped stalk of the spermathecal (SS, Fig. 9D) (vs S-shaped, Fig. 11D).

#### Description.

**Male** (Fig. 9A). Total length 8.62. Carapace 4.44 long, 3.31 wide; opisthosoma 3.99 long, 2.62 wide. Carapace greyish brown with distinct light median band. Eye sizes and interdistances: AME 0.13, ALE 0.13, PME 0.35, PLE 0.30; AME–AME 0.10, AME–ALE 0.08, PME–PME 0.30, PME–PLE 0.40. Clypeus 0.24 high. Leg measurements: I 11.11 (3.09, 4.08, 2.60, 1.34); II 10.59 (2.88, 3.87, 2.47, 1.37); III 10.88 (3.05, 3.63, 2.91, 1.29); IV 15.24 (3.89, 4.99, 4.60, 1.76).

***Palp*** (Fig. 8). Tegular apophysis strong, finger-shaped. Terminal apophysis strong and triangle-like, under the embolus. Embolus needle-shaped, with abrupt tip. Retrolateral tegular apophysis with two arms (digital-like anterior arm and hook-like posterior arm) and with a groove on the dorsal surface (maybe functioning as conductor).

**Female** (Fig. 9B). Total length 9.20. Carapace 3.96 long, 3.05 wide; opisthosoma 5.21 long, 3.51 wide. Carapace brown with light stripe behind cephalic part and wide band anteriorly, light band with pair of dark dots and longitudinal stripe. Eye sizes and interdistances: AME 0.15, ALE 0.15, PME 0.35, PLE 0.29; AME–AME 0.12, AME–ALE 0.08, PME–PME 0.30, PME–PLE 0.37. Clypeus 0.33 high. Leg measurements: I 9.90 (2.87, 3.60, 2.28, 1.15); II 7.76 (2.24, 2.71, 1.80, 1.01); III 9.89 (2.68, 3.28, 2.42, 1.51); IV 12.88 (3.49, 4.30, 3.87, 1.22).

***Epigyne*** (Figs 9C, D). Septum under epigynal plate. Copulatory openings semicircular, located on shoulders of septum. Stalk of spermatheca C-shaped, head of spermatheca spherical. Fertilization ducts crescent-shaped.

#### Distribution.

China (Anhui, Zhejiang) (Fig. 16).

### ﻿ Kuncosa zhui

Taxon classificationAnimaliaAraneaeLycosidae

(Yu & Song, 1988) comb. nov.

7AEB3522-A4E9-563F-BF4E-62E237429439

Common name. 朱氏鲲蛛 Figs 1D, E , 10 , 11 , 16

Pardosa zhui Yu & Song, 1988: 30, figs 11–13 (♀); Chen and Zhang 1991: 206, fig. 209.1–3 (♀); Song et al. 1999: 335, fig. 200C (♀).
Arctosa fujiii : Chen and Song 1999: 141, figs 16–17 (♂); Song et al. 1999: 318, fig. 188N (♂). Misidentified.
Arctosa kwangreungensis : Yin et al. 1993: 11, figs 14–18 (♂♀); Yin et al. 1997b: 90, fig. 40a–e (♂♀); Song et al. 1999: 319, fig. 189D (♀); Yin et al. 2012: 799, fig. 399a–e (♂♀). Misidentified.

#### Material examined.

**China**, **Chongqing**: 27♂ 68♀, Jiangjing District, Simian Mt. Nature Reserve, Huiqianyan, 28°37'06"N, 106°22'10"E, elev. 1003 m, 28 April 2012, L.Y. Wang and X.K. Jiang leg. (SWUC) • 20♂ 54♀, Huiqianyan, 27 April 2012, L.Y. Wang and X.K. Jiang leg. (SWUC) • 1♀, Feilong Temple, 28°37'11"N, 106°22'09"E, elev. 995 m, 22 July 2011, L.Y. Wang and M.X. Liu leg. (SWUC) • 1♂ 1♀, Feilong Temple, 22 March 2012, L.Y. Wang and X.K. Jiang leg. (SWUC) • **Guizhou**: 1♀, Zunyi City, Suiyang Co., Kuankuoshui Nature Reserve, 28°13′34"N, 107°09′34"E, elev. 1461 m, 3 June 2010, Z.X. Li and L.Y. Wang leg. (SWUC) • 1♀, Kuankuoshui Nature Reserve, Rangshui, Qizhu Mt., 28°13′44"N, 107°13′23"E, elev. 1109 m, Z.X. Li and L.Y. Wang leg. (SWUC) • 1♀, Kuankuoshui Nature Reserve, 14 August 2010, Z.X. Li and Z.S. Zhang leg. (SWUC) • 2♀, Kuankuoshui Nature Reserve, 28°14′18"N, 107°07′14"E, elev. 1545 m, 15 August 2010, Z.X. Li and Z.S. Zhang leg. (SWUC) • 1♀, Shibing Co., Yuntai Mt., 27°06′48"N, 108°06′57"E, elev. 879 m, 17 July 2012, X.K. Jiang and D. Wang leg. (SWUC) • 6♂, Yuntai Mt, 18 October 2012, L.Y. Wang leg, X.K. Jiang and X.W. Meng leg. (SWUC) • 3♂ 3♀, Jiangkou Co., Fanjing Mt. Nature Reserve, Heihewan Administrative Station, 27°50′58"N, 108°46′06"E, elev. 620 m, 5 April 2013, G.C. Zhou leg. (SWUC) • 1♀, Yinjiang Co., Yangxi Town, Hexin Vill., 27°39′32"N, 108°31′15"E, elev. 665 m, 11 May 2014, L.Y. Wang and X.W. Meng leg. (SWUC) • 1♀, Yinjiang Co., Yangxi Town, Yaojiawan, 27°38′27"N, 108°33′06"E, elev. 888 m, 12 May 2014, L.Y. Wang and X.W. Meng leg. (SWUC) • 1♂ 2♀, Yinjiang Co., Yangxi Town, Weigan Vill., Lishuping, 27°41′56"N, 108°29′32"E, elev. 1016 m, 15 May 2014, L.Y. Wang and X.W. Meng leg. (SWUC) • **Hubei**: 5♂ 2♀, Badong Co., Liujiagou, 31°09′43N 110°22′48"E, elev. 287 m, 24 March 2014, L.Y. Wang leg. (SWUC) • 2♂ 2♀, Badong Co., Yesanguan Town, 30°38′01"N, 110°20′26"E, elev. 1170 m, 24 March 2014, L.Y. Wang leg. (SWUC) • **Jiangxi**: 187♂ 62♀, Ji’an City, Taihe Co., Heshi Town, Jiangbei Vill., 26°51′50"N, 114°42′05"E, elev. 61 m, 17 February 2015, L.Y. Wang and H.Y. Liu leg. (SWUC) • **Hunan**: 3♂ 2♀, Changsha City, Yuelu Mt., 28°10′30"N, 112°56′01"E, elev. 83 m, 26 October 2013, L.Y. Wang, X.K. Jiang and X.W. Meng leg. (SWUC).

#### Diagnosis.

See the diagnosis of *K.ningboensis* (Yin, Bao & Zhang, 1996).

#### Description.

**Male** (Fig. 11A). Total length 7.40. Prosoma 4.13 long, 3.17 wide; opisthosoma 3.68 long, 2.38 wide. Carapace greyish brown. Eye sizes and interdistances: AME 0.14, ALE 0.14, PME 0.31, PLE 0.30; AME–AME 0.10, AME–ALE 0.07, PME–PME 0.28, PME–PLE 0.35. Clypeus height 0.22. Leg measurements: I 10.81 (3.01, 4.10, 2.53, 1.17); II 10.27 (2.90, 3.77, 2.42, 1.18); III 10.50 (2.88, 3.57, 2.85, 1.20); IV 14.14 (3.63, 4.85, 4.22, 1.44).

***Palp*** (Fig. 10). Tegular apophysis strong, finger-shaped. Terminal apophysis strong and fingernail-like, under the embolus. Embolus needle-shaped, with abrupt tip. Retrolateral tegular apophysis with two arms (triangular-like anterior arm and hook-like posterior arm) and with a groove on the dorsal surface (maybe functioning as conductor).

**Female** (Fig. 11B). Total length 9.40. Prosoma 4.47 long, 3.31 wide; opisthosoma 5.43 long, 3.83 wide. Eye sizes and interdistances: AME 0.17, ALE 0.15, PME 0.37, PLE 0.33; AME–AME 0.13, AME–ALE 0.09, PME–PME 0.31, PME–PLE 0.41. Clypeus height 0.28. Leg measurements: I 10.46 (2.95, 3.94, 2.43, 1.14); II 9.77 (2.89, 3.45, 2.32, 1.11); III 10.26 (2.97, 3.49, 2.64, 1.16) IV 14.07 (3.77, 4.51, 4.19, 1.60).

***Epigyne*** (Figs 11C, D). Septum under epigynal plate, T-shaped. Copulatory openings slit-like, located on the shoulders of septum. Stalk of spermatheca S-shaped, head of spermatheca spherical. Fertilization ducts crescent-shaped.

#### Distribution.

China (Chongqing, Guizhou, Hubei, Hunan, Jiangxi, Jiangsu).

#### Remarks.

Yin et al. (1993) regarded the specimens collected from Hunan (Sangzhi, Chengbu, Ningxiang, Changsha) as *Arctosakwangreungensis*. Chen and Song (1999) regarded the specimens collected from Badong, Hubei as *A.fujiii*. Yu and Song (1988) described *Pardosazhui* based on the holotype from Ji’an, Jiangxi. After observing the specimens collected from Hunan, Badong, and Ji’an, and comparing them with the type specimens of *A.fujiii* and the attached figures in the description of *A.kwangreungensis* Paik & Tanaka, 1986, the specimens from these places are neither *A.fujiii* nor *A.kwangreungensis*, but *Pardosazhui*. Therefore, here we conclude that the records of *A.fujiii* and *A.kwangreungensis* in China are all misidentified and all refer to *Kuncosazhui*.

### ﻿ Loongcosa gen. nov.

Taxon classificationAnimaliaAraneaeLycosidae

8DD212E2-E668-5E0F-AA3F-298989EAA91E

https://zoobank.org/C3FC6E20-CB5E-4D06-BB1E-AA2FF0FA24CC

#### Type species.

*Pardosadentitegulum* Yin, Peng, Xie, Bao & Wang, 1997.

#### Etymology.

The generic name is a compound noun derived from the Chinese totem ‘loong’ (龙) and ‘-cosa’, a common ending in Lycosidae genera. The gender is feminine.

#### Diagnosis.

**Figure 12.**
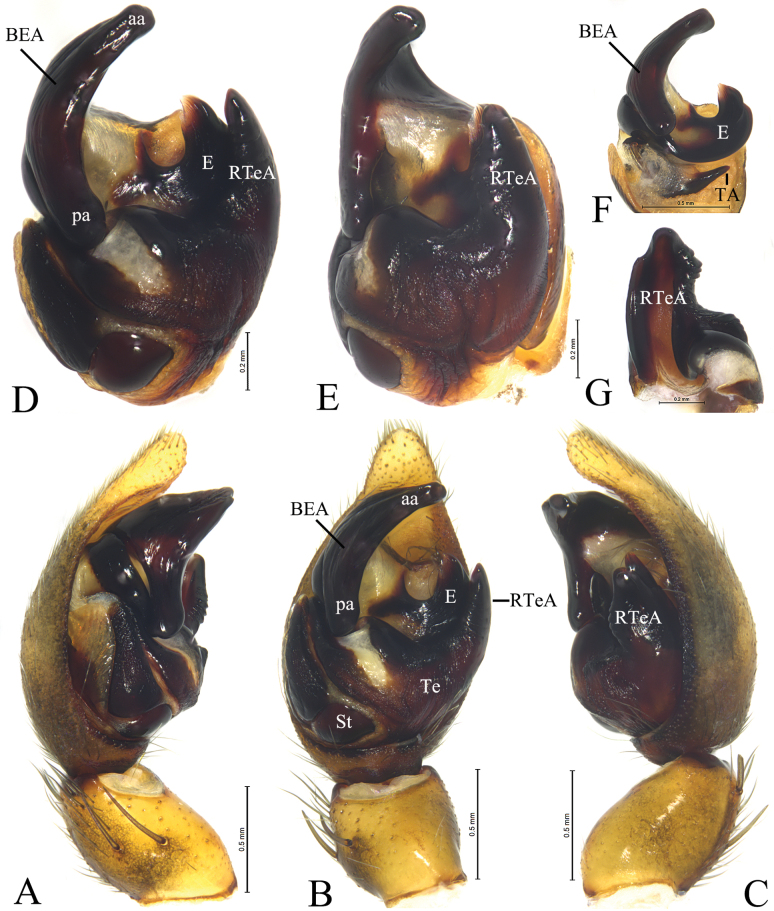
*Loongcosadentitegulum* (Yin, Peng, Xie, Bao & Wang, 1997), comb. nov. from Huaping Natural Reserve **A** male left palp, prolateral view **B** same, ventral view **C** same, retrolateral view **D** left male palp, bulb, ventral view **E** same, retrolateral view **F** embolus and terminal apophysis, ventral view **G** tegular apophysis, prolateral view. Abbreviations: aa = anterior arm of basoembolic apophysis; BEA = basoembolic apophysis; E = embolus; pa = poster ior arm of basoembolic apophysis; RTeA = retrolateral tegular apophysis; St = subtegulum; TA = terminal apophysis; Te = tegulum.

**Figure 13.**
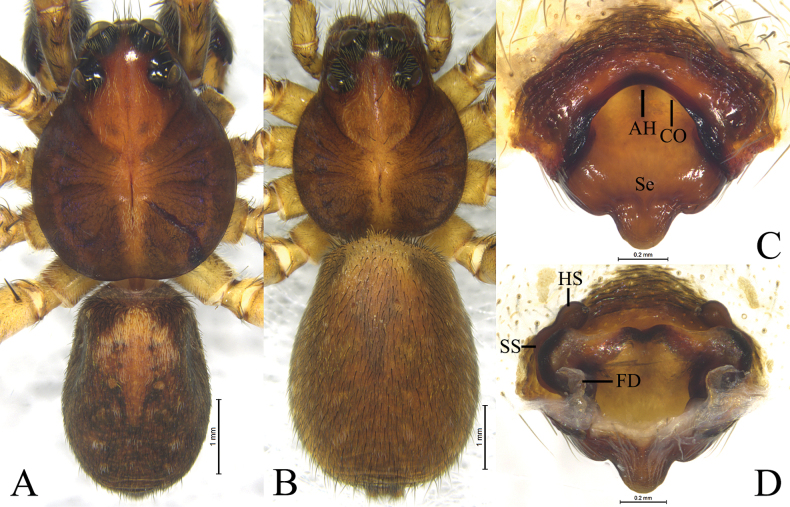
*Loongcosadentitegulum* (Yin, Peng, Xie, Bao & Wang, 1997), comb. nov. from Huaping Natural Reserve **A** male habitus, dorsal view **B** female habitus, dorsal view **C** epigyne, ventral view **D** vulva, dorsal view. Abbreviations: AH = anterior hood; CO = copulatory opening; FD = fertilization duct; HS = head of spermatheca. Se = septum; SS = stalk of spermatheca.

**Figure 14.**
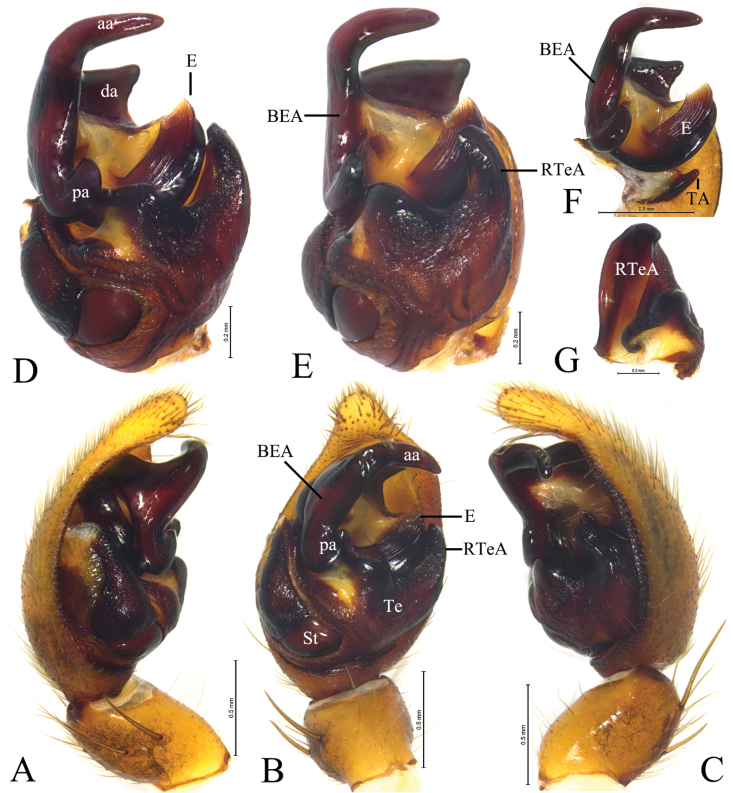
*Loongcosawuyiensis* (Yu & Song, 1988), comb. nov. from Tianmu Mountain **A** male left palp, prolateral view **B** same, ventral view **C** same, retrolateral view **D** left male palp, bulb, ventral view **E** same, retrolateral view **F** embolus and terminal apophysis, ventral view **G** tegulum apophysis, prolateral view. Abbreviations: aa = anterior arm of basoembolic apophysis; BEA = basoembolic apophysis; da = dorsal arm of basoembolic apophysis; E = embolus; pa = posterior arm of basoembolic apophysis; RTeA = retrolateral tegular apophysis; St = subtegulum; TA = terminal apophysis; Te = tegulum.

**Figure 15.**
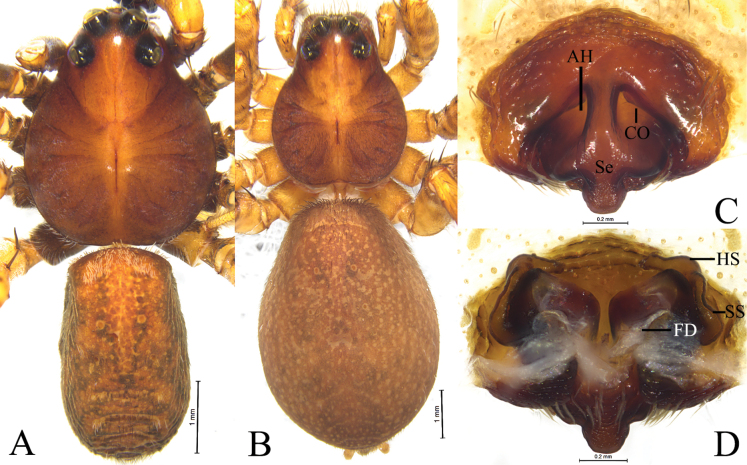
*Loongcosawuyiensis* (Yu & Song, 1988), comb. nov. from Tianmu Mountain **A** male habitus, dorsal view **B** female habitus, dorsal view **C** epigyne, ventral view **D** vulva, dorsal view. Abbreviations: AH = anterior hood; CO = copulatory opening; FD = fertilization duct; HS = head of spermatheca. Se = septum; SS = stalk of spermatheca.

*Loongcosa* gen. nov. is similar to *Houcosa* gen. nov. (Figs 2, 3) and *Kuncosa* gen. nov. (Figs 4–11) in having 2 pairs of spines on the male palpal tibia, a strong basoembolic apophysis (BEA), Figs 2, 12, 14), an extended posterior septum of the epigyne (Figs 3C, D, 13C, D, 15C, D), but differs from the latter two genera by the short tibia, 1/3 the length of the cymbium (Figs 12A–C, 14A–C) (vs as long as in *Houcosa* and as half-length in *Kuncosa*), tegular apophysis absent (TeA, Figs 12A–E, 14A–E) (vs present in both *Houcosa* and *Kuncosa*), terminal apophysis short, sclerotized, spine-like and located on posteriorly to embolus (TA, Figs 12F, 14F) (vs long, membranous, needlelike and located anteriorly to embolus in *Houcosa* and strong, triangular in *Kuncosa*), wide and heavily sclerotized embolus (Em, Figs 12B, D, F, 14B, D, F) (vs thin in both *Houcosa* and *Kuncosa*), the strong sclerotized and grooved retrolateral tegular apophysis (RTeA, Figs 12B–E, G, 14B–E, G) (vs triangular in *Houcosa* and bifurcated in *Kuncosa*), large and almost tongue-shaped septum with a kind of scape (Sc, Figs 13C, 15C) (vs small, semicircular in *Houcosa* and truncated posterior margin in *Kuncosa*), the arc-shaped stalk of the spermatheca (SS, Figs 13D, 15D) (vs S-shaped in *Houcosa*), and a small head of the spermatheca (SH, Figs 13D, 15D) (vs large and suboval in *Houcosa*).

#### Description.

Carapace brown with distinct light median band, median band wide in cephalic part, with pair of dark dots, thoracic part of median band thin (Figs 13A, B, 15A, B). Chelicerae brown, with 3 teeth on both margins. Labium brown, with dark base. Endites brown. Sternum brown, with sparse black setae. Legs brown, with black pigmentation. Leg formula: 4132. Opisthosoma oval, dorsum black brown, with yellow setae anteriorly, and 5 pairs of white markings in posterior half when live, venter brown.

***Palp*** (Figs 12, 14): Tibia ca 1.4 times longer than wide in lateral view, with 2 pairs of spines, disto-dorsal part and proximo-ventral part rounded. Lacking claws; Bulb ca 1.4 longer than wide; Tegular apophysis, conductor and median apophysis lacking. Terminal apophysis (TA) short, spine-like, sclerotized and located posterior to embolus. Basoembolic apophysis (BEA) large and heavily sclerotized, with 2 or 3 distinct arms. Embolus short, wide, heavily sclerotized, roundly bent, tip pointed. Retrolateral tegular apophysis strongly sclerotized and grooved.

***Epigyne*** (Figs 13C, D, 15C, D): Epigynal plate almost as long as wide; anterior part heavily sclerotized, fovea distinct anteriorly; anterior margin with one or pair of anterior hoods (AH), septum broad either with stalk or without, posteriorly with a kind of scape (Sc). Stalk of spermatheca C-shaped, head of spermatheca spherical or irregular.

#### Composition.

*Loongcosadentitegulum* (Yin, Peng, Xie, Bao & Wang, 1997) and *L.wuyiensis* (Yu & Song, 1988).

#### Distribution.

**Figure 16.**
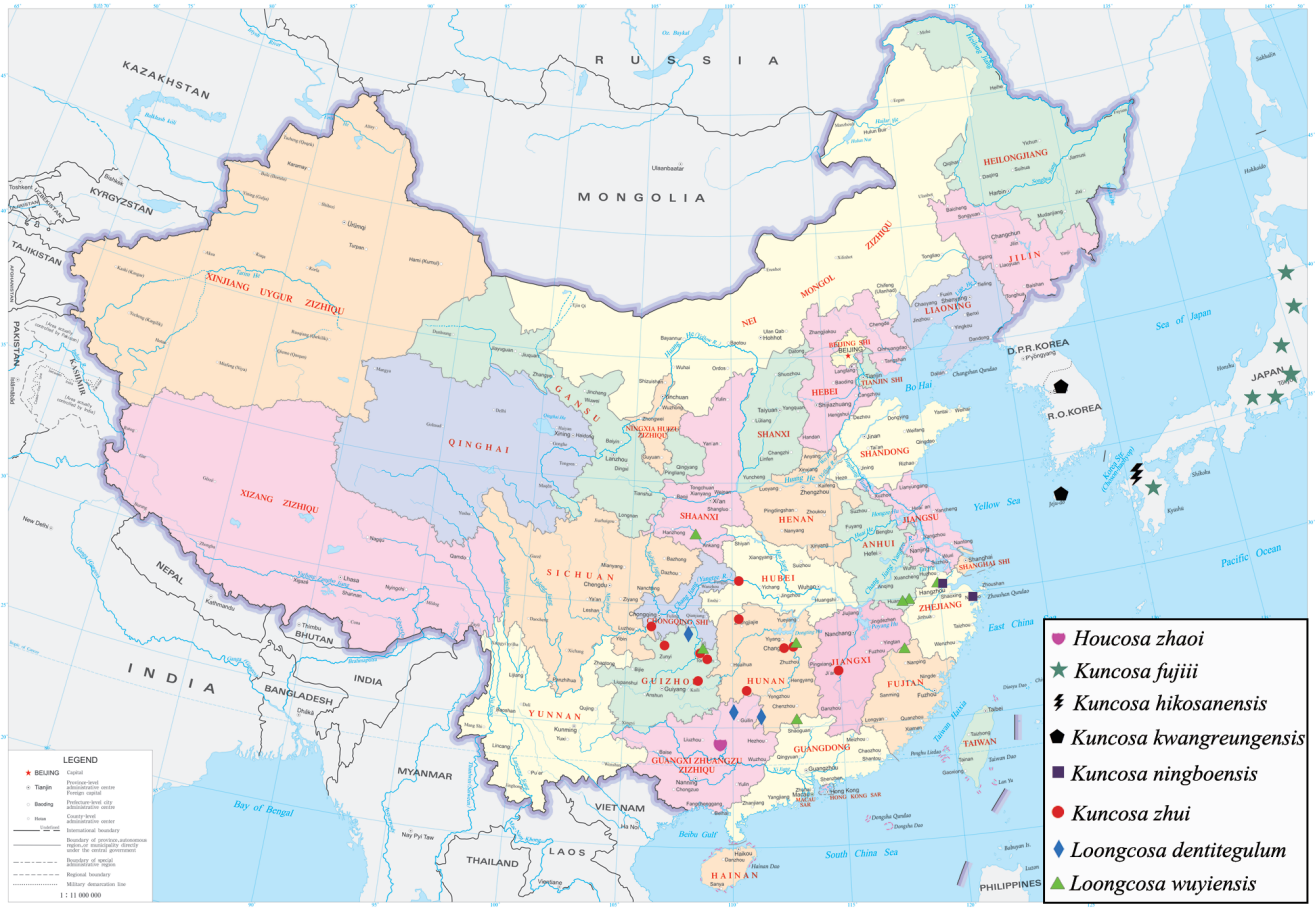
Geographic distribution of *Houcosa*, *Kuncosa* and *Loongcosa*.

China (Anhui, Fujian, Hunan, Guizhou, Guangxi, Shaanxi, Zhejiang) (Fig. 16).

### ﻿ Loongcosa dentitegulum

Taxon classificationAnimaliaAraneaeLycosidae

(Yin, Peng, Xie, Bao & Wang, 1997) comb. nov.

D6F77E0E-949F-5739-BE13-0590766AA948

Common name. 齿盾龙蛛 Figs 1F, G , 12 , 13 , 16

Pardosa dentitegulum Yin et al., 1997a: 20, figs 8–13 (♂); Yin et al. 1997b: 268, fig. 126a–f (♂); Song et al. 1999: 330, fig. 194S (♂); Yin et al. 2012: 838, fig. 419a–f (♂).

#### Material examined.

**China**: 4♂ 2♀, Guangxi, Longsheng Co., Sanmen Town, Huaping National Nature Reserve, 25°37′54"N, 109°54′30"E, elev. 555 m, 30 April 2023, L.Y. Wang and Q.L. Lu leg. (SWUC) • 2♂ 5♀, Guangxi, Longsheng Co., Sanmen Town, Huaping National Nature Reserve, 25°36′19"N, 109°54′21"E, elev. 880 m, 1 May 2023, L.Y. Wang and Q.L. Lu leg. (SWUC) • 1♂ 1♀, Guizhou, Wuchuan Co., Hongsi Township, Yueliang Vill., Laohuba, 28°41′11"N, 108°09′25"E, elev. 983 m, 21 April 2013, X.K. Jiang leg. (SWUC).

#### Diagnosis.

This species differs from *L.wuyiensis* by the short and C-shaped basoembolic apophysis with 2 arms in ventral view (BEA, Fig. 12B, D, F) (vs long, hook-shaped with 3 arms, Fig. 14B, D–F), epigyne with an anterior hood (AH, Fig. 13C) (vs with 2 hoods, Fig. 15C), and septum lacking stalk (Fig. 13C) (vs having a stalk, Fig. 15C).

#### Description.

**Male** (Fig. 13A). Total length 6.38. Carapace 3.48 long, 2.77 wide; opisthosoma 2.91 long, 1.99 wide. Carapace greyish brown with distinct light median band and submarginal stripes, median band wide in cephalic part, with pair of dark dots, thoracic part of median band thin. Eye sizes and interdistances: AME 0.12, ALE 0.13, PME 0.41, PLE 0.33; AME–AME 0.13, AME–ALE 0.07, PME–PME 0.31, PME–PLE 0.32. Clypeus height 0.21. Leg measurements: I 8.41 (2.34, 3.01, 2.11, 0.95); II 8.05 (2.25, 2.72, 2.10, 0.98); III 8.28 (2.26, 2.65, 2.33, 1.04); IV 11.64 (2.79,3.71, 3.81, 1.33).

***Palp*** (Fig. 12): Basoembolic apophysis with 2 arms, anterior (aa) and posterior (pa), almost as long as tegulum. Embolus broad, short and arc-shaped. Terminal apophysis short, sclerotized and located posterior to embolus. Retrolateral tegular apophysis sclerotized, twice as long as wide, with a groove on the dorsal surface.

**Female** (Fig. 13B). Total length 7.25. Carapace 3.42 long, 2.75 wide; opisthosoma 3.91 long, 2.99 wide. Colouration as in male. Eye sizes and interdistances: AME 0.13, ALE 0.13, PME 0.41, PLE 0.35; AME–AME 0.12, AME–ALE 0.09, PME–PME 0.33, PME–PLE 0.36. Clypeus height 0.15. Leg measurements: I 8.92 (2.54, 3.25, 2.12, 1.01); II 8.44 (2.54, 3.11, 1.87, 0.92); III 8.86 (2.68, 2.60, 2.36, 1.22); IV 12.18 (2.90, 4.01, 3.74, 1.53).

***Epigyne*** (Fig. 13C, D). Epigynal plate almost as long as wide, anterior hood (AH) unpaired. Septum originated beneath hood. Copulatory openings semicircular. Stalk of spermatheca C-shaped, head of spermatheca spherical, ca 1.7 times wider than stalk.

#### Distribution.

China (Hunan, Guizhou, Guangxi) (Fig. 16).

### ﻿ Loongcosa wuyiensis

Taxon classificationAnimaliaAraneaeLycosidae

(Yu & Song, 1988) comb. nov.

60FDB4D5-DD28-5D2E-A4FA-3CDFCBF64195

Common name. 武夷龙蛛 Figs 1H, I , 14 , 15 , 16

Pardosa wuyiensis Yu & Song, 1988: 36, figs 35–38 (♂♀); Chen and Zhang 1991: 202, fig. 203. 1–4 (♂♀); Yin et al. 1997b: 271, fig. 128a–g (♂♀); Song et al. 1999: 335, fig. 199F, N (♂♀); Yin et al. 2012: 860, fig. 431a–g (♂♀); Dong 2018: 64, figs 9–7. A–F, pl. 14 (♂♀).

#### Material examined.

**China**, **Anhui**: 1♂ 1♀, Huangshan City, Xiuning Co., Qiyun Mt., Fanglazhai, 29°48′11"N, 118°02′02"E, elev. 478 m, X.K. Jiang and L.Y. Wang leg. (SWUC) • 1♂, Xuancheng City, Jixi Co., Fuling Town, Yonglai Vill., 30°08′43"N, 118°50′54"E, elev. 718 m, 3 June, 2013, F. Zhang leg. (SWUC) • **Fujian**: 21♂ 28♀, Wuyi Mountain Natural Reserve, Moshikeng, 27°48′46"N, 117°53′02"E, elev. 1154 m, 20 May 2004, F. Zhang leg. (SWUC) • **Guizhou**: 44♂ 51♀, Guizhou, Yinjiang Co., Yangxi Town, Weigan Vill., Lishuping, 27°41′47"N, 108°29′33"E, elev, 1016 m, 15 May 2014, L.Y. Wang and X.W. Meng leg. (SWUC) • **Shaanxi**: 1♀, Hanzhong City, Xixiang Co., Yankou Town, Wuzi Mt., 32°56′50"N, 107°50′55"E, elev. 787 m, 27 May 2013, M.X. Liu and X.W. Meng leg. (SWUC) • **Zhejiang**: 5♂ 16♀, Hangzhou City, Lin’an Co., Tianmu Mt., 30°18′51"N, 119°26′25"E, elev. 346 m, 21–23 April 2011, Z.X. Li and L.Y. Wang leg. (SWUC) • 1♀, Tianmu Mt., 30°18′43"N, 119°26′53"E, elev. 335 m, 9 April 2012, L.Y. Wang leg. (SWUC) • 1♀, Tianmu Mt., near Chanyuan Temple, 30°19′10"N, 119°26′52"E, elev. 382 m, 9 April 2012, L.Y. Wang leg. (SWUC).

#### Diagnosis.

See the diagnosis of *L.dentitegulum* (Yin, Peng, Xie, Bao & Wang, 1997).

#### Description.

**Male** (Fig. 15A). Total length 6.25. Carapace 3.29 long, 2.67 wide; opisthosoma 2.93 long, 1.79 wide. Carapace greyish brown, with median band, submarginal stripe absent. Opisthosoma lacking distinct cardiac mark. Eye sizes and interdistances: AME 0.11, ALE 0.11, PME 0.36, PLE 0.34; AME–AME 0.11, AME–ALE 0.08, PME–PME 0.31, PME–PLE 0.31. Clypeus 0.18 high. Leg measurements: I 8.11 (2.17, 2.90, 2.00, 1.04); II 7.80 (2.15, 2.68, 1.97, 1.00); III 7.96 (2.15, 2.56, 2.20, 1.05); IV 11.33 (2.87, 3.64, 3.48, 1.34).

***Palp*** (Fig. 14). Basoembolic apophysis with three arms: dorsal (da), posterior (pa), and anterior (aa). Embolus (E) very broad, terminal part almost straight, ca 2 times longer than wide, tip abrupt. Terminal apophysis short, sclerotized, and located on the posterior side of the embolus. Retrolateral tegular apophysis sclerotized, twice as long as wide, with a groove on the dorsal surface.

**Female** (Fig. 15B). Total length 9.09. Carapace 3.59 long, 2.80 wide; opisthosoma 5.25 long, 3.82 wide. Colouration as in male. Eye sizes and interdistances: AME 0.11, ALE 0.11, PME 0.44, PLE 0.35; AME–AME 0.13, AME–ALE 0.09, PME–PME 0.36, PME–PLE 0.35. Clypeus height 0.20. Leg measurements: I 8.34 (2.41, 3.07, 1.87, 0.99); II 7.99 (2.32, 2.83, 1.88, 0.96); III 8.04 (2.25, 2.55, 2.21, 1.03); IV 11.42 (2.83, 3.73, 3.51, 1.35).

***Epigyne*** (Fig. 15C, D). Epigynal plate ca 1.3 times wider than long, fovea distinct, septum with distinct stalk. Stalk of spermatheca C-shaped, head of spermatheca elongate.

#### Distribution.

China (Anhui, Fujian, Hunan, Guizhou, Shaanxi, Zhejiang) (Fig. 16).

## ﻿Discussion

The three new genera established in this paper have many common characteristics: they live on forest floors, the tibia of the male palps with 2 pairs of spines, with a large and heavily sclerotized basoembolic apophysis, and a sclerotized retrolateral tegular apophysis (triangular in *Houcosa*, bifurcated in *Kuncosa*, heavily sclerotized and grooved in *Loongcosa*), no conductor and median apophysis. The epigyne is strongly sclerotized, the stalk of the spermatheca is mostly S-shaped, and the spermatheca is relatively small. In addition, in Piacentini and Ramírez (2019), the genus *Kuncosa* gen. nov. (*Arctosakwangreungensis* Paik & Tanaka, 1986) and the genus *Hygrolycosa* Dahl, 1908 (*Hygrolycosarubrofasciata* (Ohlert, 1865)) are clustered together and are jointly classified into the subfamily Sosippinae. However, the differences in the palps between this new genus and the subfamily Sosippinae are quite significant; as such Sosippinae are web-building spiders, whereas these three new genera are all wandering hunters. Therefore, the subfamily placement of these three new genera is currently unknown.

## Supplementary Material

XML Treatment for Houcosa

XML Treatment for Houcosa zhaoi

XML Treatment for Kuncosa

XML Treatment for Kuncosa fujiii

XML Treatment for Kuncosa hikosanensis

XML Treatment for Kuncosa ningboensis

XML Treatment for Kuncosa zhui

XML Treatment for Loongcosa

XML Treatment for Loongcosa dentitegulum

XML Treatment for Loongcosa wuyiensis

## ﻿Additional information

### Author ORCIDs

Lu-Yu Wang https://orcid.org/0000-0002-5250-3473

Yuri M. Marusik https://orcid.org/0000-0002-4499-5148

Zhi-Sheng Zhang https://orcid.org/0000-0002-9304-1789

### Data availability

All of the data that support the findings of this study are available in the main text.
